# SIRT3 Protects Against Acute Kidney Injury via AMPK/mTOR-Regulated Autophagy

**DOI:** 10.3389/fphys.2018.01526

**Published:** 2018-11-14

**Authors:** Wenyu Zhao, Lei Zhang, Rui Chen, Hanlan Lu, Mingxing Sui, Youhua Zhu, Li Zeng

**Affiliations:** Department of Organ Transplantation, Changhai Hospital, Second Military Medical University, Shanghai, China

**Keywords:** cecal ligation and puncture, sirtuin 3, acute kidney injury, AMPK/mTOR pathway, autophagy

## Abstract

Acute kidney injury (AKI), which involves the loss of kidney function caused by damage to renal tubular cells, is an important public health concern. We previously showed that sirtuin (SIRT)3 protects the kidneys against mitochondrial damage by inhibiting the nucleotide-binding domain (NOD)-like receptor protein 3 (NLRP3) inflammasome, attenuating oxidative stress, and downregulating proinflammatory cytokines. In this article, we investigated the role of autophagy, mediated by a mammalian target of rapamycin (mTOR) and AMP-activated protein kinase (AMPK), in the protective effect of SIRT3, against sepsis-induced AKI, in a mouse model of cecal ligation and puncture (CLP). The AKI in CLP mice was associated with the upregulation of autophagy markers; this effect was abolished in SIRT3^−^ mice in parallel with the downregulation of phospho (p)-AMPK and the upregulation of p-mTOR. Pretreatment with the autophagy inhibitor 3-methyladenine (3-MA) or AMPK inhibitor compound isotonic saline (C), exacerbated AKI. SIRT3 overexpression promoted autophagy, upregulated p-AMPK and downregulated p-mTOR in CLP mice, attenuating sepsis-induced AKI, tubular cell apoptosis, and inflammatory cytokine accumulation in the kidneys. The blockage of autophagy induction largely abolished the protective effect of SIRT3 in sepsis-induced AKI. These findings indicate that SIRT3 protects against CLP-induced AKI by inducing autophagy through regulation of the AMPK/mTOR pathway.

## Introduction

Acute kidney injury (AKI) is a loss of kidney function, with mixed etiology, including sepsis, ischemia, and nephrotoxicity (Makris and Spanou, 2016). AKI is classified as follows: pre-renal AKI, acute post-renal obstructive nephropathy, and intrinsic acute kidney diseases. AKI is an important public health concern and acute renal failure occurs in approximately 17% of patients in intensive care units (ICUs), of whom, 50% develop renal failure during hospitalization (Clermont et al., 2002). The mortality rate of ICU patients with renal failure is 23%. Mitochondrial injury is a hallmark of AKI, and mitochondrial function is partly regulated by sirtuins (SIRTs), a conserved family of nictotinamide adenine dinucleotide (NAD^+^)-dependent deacetylases, involved in several processes, such as stress response, metabolism, development, and longevity (Haigis and Sinclair, 2010). SIRT1–7 has been identified in mammals, and SIRT3–5 is mitochondrial (Lombard, et al., 2011). SIRT3 is a potent deacetylase, expressed in humans as an enzymatically inactive 44-kDa protein with an N-terminal mitochondrial targeting sequence. SIRT3 is proteolytically processed to a mature 28-kDa active enzyme that functions as a positive regulatorof mitochondrial activity by activating and deacetylating the components of the electron transport chain and acetyl-CoA synthetase (ACS) (Onyango et al., 2002; Schwer et al., 2002). SIRT3 is also involved in the defense against oxidative stress, and its suppression of intracellular reactive oxygen species (ROS) levels, may mediate its promotion of cell survival (Kong et al., 2010; Lombard et al., 2011).

Autophagy, a self-degradation process, by which, damaged organelles and macromolecules are degraded and recycled to maintain cellular homeostasis, is associated with the pathogenesis of AKI (Kaushal and Shah, 2016). Autophagy is induced in response to cellular stressors such as starvation, hypoxia, nutrient and growth factor deprivation, and oxidative injury, which are associated with AKI (Kundu and Thompson, 2008; Kroemer and Marino, 2010). The production of ROS and oxidative stress, which are involved in AKI, trigger autophagy in mammalian cells and tissues and ROS, that can damage mitochondria, which are abundant in the kidneys (Chen et al., 2009; Scherz-Shouval and Elazar, 2011). Autophagy modulation, in response to stress, is mediated by kinases, such as mammalian target of rapamycin (mTOR) and AMP-activated protein kinase (AMPK); these kinases may be involved in the development of autophagy in AKI (Kim et al., 2011; Dunlop and Tee, 2014). The AMPK, an energy sensor, that regulates cellular metabolism and homeostasis, promotes autophagy, whereas mTOR, a serine/threonine protein kinase that regulates cell growth, proliferation, motility, and survival, is an inhibitor of autophagy (Alers et al., 2012).

The autophagy-related protein, BECN1, an interacting partner of the antiapoptotic protein, B-cell lymphoma 2 (Bcl-2), plays a role in the formation of autophagosomes, in addition to microtubule-associated protein, I light chain 3 (LC3-I). The LC3-I is conjugated to phosphatidylethanolamine (PE) to generate LC3-II, which is recruited to the autophagosomal membranes and degraded after fusion of autophagosomes to lysosomes (Tanida et al., 2008).

We have previously shown that SIRT3 protects against mitochondrial damage in the kidneys by attenuating ROS production, inhibiting the NRLP3 inflammasome, attenuating oxidative stress, and downregulating interleukin (IL)-1β and IL-18 (Zhao et al., 2016). In this study, we further examined the role of SIRT3 in the mitochondrial damage associated with AKI in a cecal ligation and puncture (CLP) model of sepsis-induced AKI, in SIRT3 knockout mice. Our results suggested that the protective role of SIRT3 in the kidney was mediated by induction of autophagy through the AMPK/mTOR pathway.

## Materials and Methods

### Animal Model of AKI

Male C57BL/6 mice (8–10 weeks old) were purchased from Shanghai Laboratory, Animal Center (Shanghai, China); 10-week-old male 129sv-SIRT3 knockout (KO) and wild-type (WT) littermate mice were purchased from The Jackson Laboratory (Bar Harbor, ME, United States). All experiments were performed in accordance with Chinese legislation on the use and care of laboratory animals and were approved by the Animal Care and Use Committee of Shanghai Hospital. The CLP was performed as described previously (Zhao et al., 2016). The cecum of mice was ligated at the colon juncture, punctured, and laced back in the abdomen before closing the incision. The Sham-operated mice were exposed to the same procedure but without ligation and puncture of the cecum. The blood isolated from mice, 24 h after CLP, was stored overnight at 4°C, the serum was isolated by centrifugation, and frozen at −80°C, until analysis. The kidney tissues were harvested, fixed, snap frozen, and stored at −80°C, until analysis.

### Experimental Design

The WT and SIRT3^−/−^ mice (*n* = 8 each) were randomly assigned to four groups as follows: Sham group: mice receiving sham operation; CLP group: mice receiving CLP operation with isotonic saline (C) treatment for supplementary fluid loss during surgery; CLP+3-methyladenine (3-MA) group: CLP mice were treated with autophagy inhibitor 3-MA (Sigma-Aldrich, St. Louis, MO, United States; 30 mg/kg) intraperitoneally (i.p.) at 12.00 and 1 h before CLP; and CLP+ComC group: CLP mice were treated with AMPK inhibitor compound C (ComC; Sigma-Aldrich; 20 mg/kg) i.p. at 12.00 and 1 h before CLP.

Male C57BL/6 mice (*n* = 6 for sham operation and CLP) were randomly assigned to four groups as follows: Control group: mice receiving sham or CLP operation; Vector group: mice were injected intravenously (i.v.) via the tail vein with 100 μl of proliferator-activated receptor γ coactivator (pGC)-FU vector lentivirus plasmids at 2 weeks and 24 h before CLP or sham surgery; pSIRT3 group: mice were injected i.v. via the tail vein with 100 μl of SIRT3 expressing lentivirus plasmids at 2 weeks and 24 h before CLP or sham surgery; and pSIRT3+3-MA group: pSIRT3-injected mice were treated i.p. with 30 mg/kg 3-MA at 12.00 and 1 h before CLP.

The blood was isolated by intracardiac puncture (ICP) and the kidneys were harvested at 24 h in the sham group or after CLP.

### Lentiviral Constructs and Production

Lentiviral vectors were constructed by amplifying the complementary DNA (cDNA) of SIRT3 by polymerase chain reaction (PCR) using the following primers: forward, 5′-TACTTCCTTCGGCTGCTTCA-3′; reverse, 5′-AAGGCGAAATCAGCCACA-3′. The PCR fragments and the pGC-FU plasmid (GeneChem, Shanghai, China) were digested with *Age*I and then ligated with T4 DNA ligase to produce pGC-FU-SIRT3. Mice were injected i.v. via the tail vein with 100 μl pGC-FU vector (Vector) or the vector expressing pSIRT3 lentiviral plasmids at 2 weeks and 24 h before CLP. The kidney tissues and blood samples were collected at 24 h after CLP.

### Measurement of Renal Function and Proinflammatory Cytokines

Renal function was analyzed by measuring blood urea nitrogen (BUN) and serum creatinine (SCr) levels using the BUN Assay Kit (Jiancheng Biotech, Nanjing, China) and the Creatinine Assay Kit (Biosino Bio-Technology). The levels of the proinflammatory cytokines, tumor necrosis factor (TNF)-α and interleukin-1β (IL-1β) were measured in kidney tissues, homogenized in phosphate-buffered saline (PBS) containing 0.05% Tween 20, by ELISA (Quantikine Kit; R&D Systems, Minneapolis, MN, United States).

### Western Blot Analysis

The kidney tissues were homogenized in a radioimmunoprecipitation assay (RIPA)buffer, which includes 1% NP40, 0.1% SDS, 100 mg/ml phenylmethylsulfonyl fluoride (PMSF), 1% protease inhibitor cocktail, and 1% phosphatase I and II inhibitor cocktail (Sigma-Aldrich), on ice. After centrifugation at 13,000 × *g* at 4°C for 30 min, protein concentration in the supernatants was determined using the bicinchoninic acid (BCA) protein assay. Equal amounts of protein were separated by 10 or 15% sodium dodecyl sulfate (SDS-PAGE) and transferred onto polyvinylidene difluoride (PVDF) membranes. The membranes were exposed to primary antibodies against SIRT3, LC3-I/II, BECN1, cleaved caspase-3, β-actin (Cell Signaling, Danvers, MA, United States), phospho (p)-AMPK (Thr172), AMPK, p-mTOR (Ser2448), and mTOR (Santa Cruz Biotechnology, Dallas, TX, United States).

### Histology and Tubular Injury Score

Formalin-fixed and paraffin-embedded kidney tissues were cut into 3-μm sections, stained with hematoxylin and eosin (H&E), and visualized under an optical microscope (Olympus Optical, Tokyo, Japan). Tubular injury was scored as follows: 0 = normal histology; 1 = tubular cell swelling, brush border loss, nuclear condensation, and up to one-third nuclear loss; 2 = as in 1, but more than one-third and less than two-thirds of nuclear loss in tubules; and 3 = more than two-thirds of nuclear loss. Each group randomly selected three fields of vision, blinded by two researchers.

### TUNEL Assay

Apoptosis was detected using the transferase terminal UTP nick end labeling (TUNEL) assay in paraffin-embedded kidney sections, using an *in situ* apoptosis detection kit (Promega, Madison, WI, United States). The TUNEL-positive cells were counted in 12 randomly selected fields from each slide at a magnification of 400×, and the percentage of TUNEL-positive cells was calculated from six kidney sections of different mice.

### Immunohistochemistry Staining

Tissue slides were blocked with goat serum at room temperature for 15 min and incubated with anti-F4/80 antibody (Santa Cruz Biotechnology) at 4°C, overnight. Slides were washed in PBS and incubated with horseradish-peroxidase (HRP)-conjugated secondary antibodies for 30 min at 37°C. Staining was visualized by reaction with diaminobenzidine tetrahydrochloride (DAB) and counterstaining with hematoxylin, and stained sections were observed under a light microscope (DP73; Olympus).

### Statistical Analysis

The data are expressed as the mean ± structural equational modeling (SEM). Results were analyzed using GraphPad Prism version 5 software (GraphPad Software, La Jolla, CA, United States). One-way analysis of variance (*P* < 0.05) or *t*-test was used to determine significant differences between groups. Individual comparisons were performed by Tukey’s hydroxysteroid dehydrogenase (HSD) *post hoc* test. The statistical significance was set at *P* < 0.05.

## Results

### SIRT3 Deletion Exacerbates Kidney Dysfunction and Inhibits Autophagy

**FIGURE 1:**
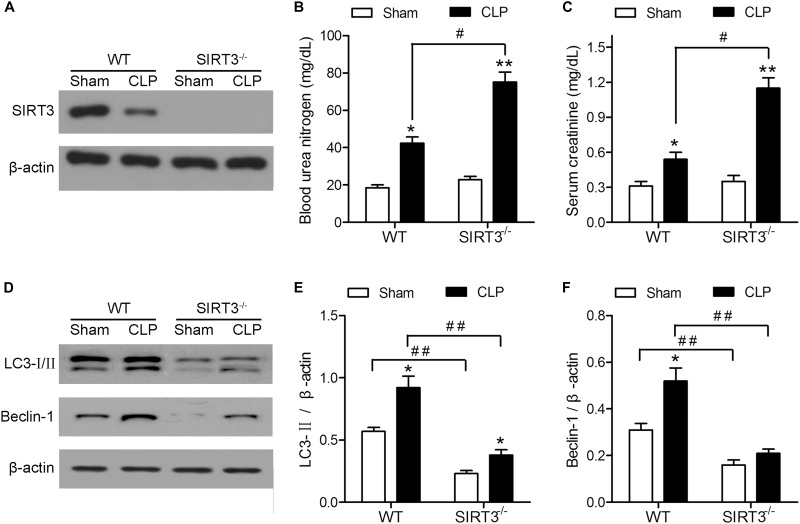
The protective effect of sirtuin (SIRT)3 against sepsis-induced kidney damage involves regulation of autophagy. The wild-type (WT) and SIRT3^−/−^ mice received cecal ligation and puncture (CLP), and sham-operated animals served as negative controls (Sham). The kidney tissues and blood samples were collected at 24 h after CLP. **(A)** The SIRT3 expression in kidney tissues was analyzed by western blotting. **(B)** Blood urea nitrogen (BUN) and **(C)** serum creatinine (SCr) levels were analyzed using commercial kits. **(D)** Western blotting of LC3-I/II and BECN1 in the whole kidney. **(E,F)** Relative protein levels were determined after normalization to β-actin. All data were expressed as the mean ± SEM (*n* = 8 mice/group). ^∗^*P* < 0.05, ^∗∗^*P* < 0.01 versus Sham in WT or SIRT3^−/−^ mice. ^#^*P* < 0.05, ^##^*P* < 0.01.

The effect of CLP on kidney function was investigated in WT and SIRT3^−/−^ KO mice by assessing the levels of BUN and creatinine. Western blotting confirmed the deletion of SIRT3 in kidney tissues collected from mice exposed to CLP or Sham operation (Figure 1A). The CLP increased the levels of BUN and creatinine in WT mice, and this increase was significantly exacerbated in SIRT3^−/−^ mice, which showed a twofold increase in the BUN and creatinine levels compared with the WT CLP mice (Figures 1B,C). Western blotting showed that CLP upregulated the autophagy markers, LC3-II and BECN1, in WT mice, and this effect was abolished by SIRT3 deletion (Figure 1D). Quantification of the autophagy markers, LC3-II and BECN1, showed significantly lower levels in SIRT3^−/−^ mice; CLP-induced upregulation of LC3-II and BECN1 was significantly inhibited in SIRT3^−/−^ mice (Figures 1E,F). These results suggest that the protective effect of SIRT3 involves regulation of autophagy.

### SIRT3 Protects Against Sepsis-Induced AKI by Regulating Autophagy

**FIGURE 2:**
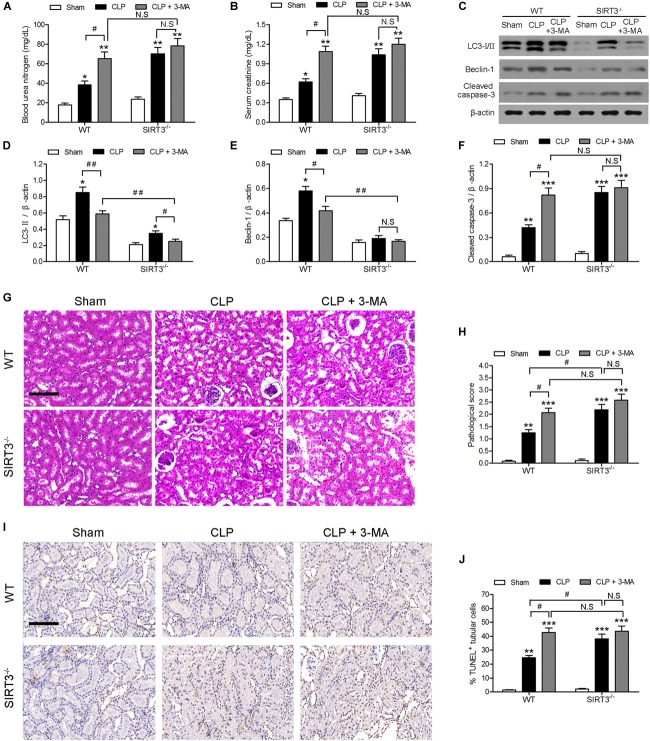
Role of autophagy in the protective effect of sirtuin (SIRT)3 against sepsis-induced acute kidney injury. Wild-type (WT) and SIRT3^−/−^ mice were injected intraperitoneally (i.p.) with either 3-methyladenine (30 mg/kg, CLP + 3-MA) or C in equivalent volumes at 12.00 and 1 h before cecal ligation and puncture (CLP). Sham-operated animals served as negative controls (Sham). Kidney tissues and blood samples were collected at 24 h after CLP using the following analysis: **(A)** Blood urea nitrogen (BUN) and **(B)** serum creatinine (SCr) were analyzed using commercial kits, **(C)** western blotting of LC3-I/II, Beclin-1 (BECN1) and cleaved caspase-3 in the whole kidney, **(D–F)** relative protein levels were determined after normalization to β-actin. **(G)** The collected kidneys of mice were stained with hematoxylin and eosin. Scale bars: 50 μm. **(H)** Quantitative evaluation of morphological tubular damage. **(I,J)** Apoptosis of renal tubular cells of mice was measured by the terminal UTP nick end labeling (TUNEL) assay and quantified. Scale bars: 50 μm. All data were expressed as the mean ± SEM (*n* = 8 mice/group). ^∗^*P* < 0.05, ^∗∗^*P* < 0.01, ^∗∗∗^*P* < 0.001 versus Sham in WT or SIRT3^−/−^ mice. ^#^*P* < 0.05, ^##^*P* < 0.01.

The involvement of autophagy in the effect of SIRT3 on sepsis-induced kidney injury was further examined using the autophagy inhibitor 3-methyladenine (3-MA). The 3-MA significantly increased the CLP-induced BUN and creatinine levels by approximately 1.5-fold in WT mice, whereas the increase was not significant in SIRT3^−/−^ mice. There was no significant difference in BUN and creatinine between WT and SIRT3^−/−^ mice under CLP+3MA conditions (Figures 2A,B). The inhibition of autophagy suppressed the CLP-induced upregulation of LC3II and BECN1 in WT mice, and this difference was reduced or not significant in KO mice (Figures 2C–E). The inhibition of autophagy increased the CLP-induced upregulation of the apoptosis marker cleaved caspase-3 by approximately twofold in WT mice. The levels of cleaved caspase-3 were not significantly affected by CLP and CLP+3-MA in the absence of SIRT3 (Figure 2F). The quantification of morphological tubular damage showed that the pathological score was significantly higher in SIRT3^−/−^ mice exposed to CLP than in WT mice, and this effect was enhanced by autophagy inhibition. In contrast, there was no significant difference in the pathological score between WT and SIRT3^−/−^ under CLP+3MA conditions (Figures 2G,H). The difference in the abundance of TUNEL^+^ cells between WT and SIRT3 KO mice under CLP conditions was 15%. There was no significant difference in the abundance of TUNEL^+^ cells between WT and SIRT3 KO mice under CLP+3MA conditions, suggesting that the relationship between SIRT3 and apoptosis was mediated by autophagy (Figures 2I,J).

### SIRT3 Protects Against Sepsis-Induced AKI by Modulating AMPK/mTOR-Mediated Autophagy

**FIGURE 3:**
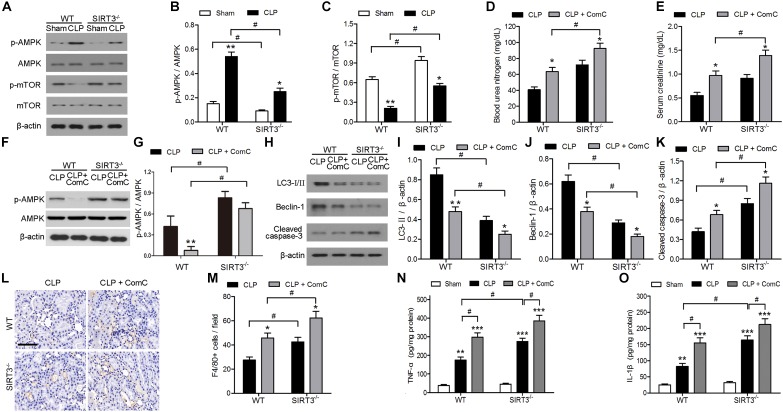
Role of AMP-activated protein kinase (AMPK) and mammalian target of rapamycin (mTOR)-mediated autophagy in the effect of sirtuin (SIRT)3 against sepsis-induced acute kidney injury. **(A–C)** Wild-type (WT) and SIRT3^−/−^ mice received cecal ligation and puncture (CLP), and sham-operated animals served as negative controls (Sham). Kidney tissues were collected at 24 h after CLP. Expression and phosphorylation of AMPK and mTOR were detected by western blotting **(A)** and normalized to AMPK **(B)** or mTOR **(C)**. All data were expressed as the mean ± SEM (*n* = 8 mice/group). ^∗^*P* < 0.05, ^∗∗^*P* < 0.01 versus Sham in WT or SIRT3^−/−^ mice. ^#^*P* < 0.05. **(D–O)** WT or SIRT3^−/−^ mice were treated intraperitoneally with either mg/kg compound C (CLP+ComC) or C in equivalent volumes at 12.00 and 1 h before CLP. Sham-operated animals served as negative controls (Sham). Kidney tissues and blood samples were collected at 24 h after CLP. **(D)** Blood urea nitrogen (BUN) and **(E)** serum creatinine were analyzed using commercial kits. **(F)** Expression and phosphorylation of AMPK and quantification of AMPK. **(G,H)** Western blotting of LC3-I/II, Beclin-1 (BECN1), and cleaved caspase-3 in the whole kidney. **(I–K)** Relative protein levels were determined after normalization to β-actin. **(L)** F4/80 expression was detected using immunohistochemical staining. Scale bars: 25 μm. **(M)** Number of F4/80^+^ cells per field. All data were expressed as the mean ± SEM (*n* = 8 mice/group). ^∗^*P* < 0.05, ^∗∗^*P* < 0.01 versus CLP in WT or SIRT3^−/−^ mice. ^#^*P* < 0.05. Levels of tumor necrosis factor (TNF)-α **(N)** and interleukin (IL)-1β **(O)** were measured by ELISA in the kidney homogenate. All data were expressed as the mean ± SEM (*n* = 8 mice/group). ^∗∗^*P* < 0.01, ^∗∗∗^*P* < 0.001 versus Sham in WT or SIRT3^−/−^ mice. ^#^*P* < 0.05.

The mechanism underlying the protective effect of SIRT3 through modulation of autophagy was further investigated by assessing the levels of the autophagy regulators AMPK and mTOR in kidney tissues exposed to sepsis-induced AKI. Western blotting and quantification of the results showed that CLP upregulated p-AMPK and downregulated p-mTOR. In SIRT3^−/−^, the threefold increase in p-AMPK in WT mice was reduced twofold, and the 0.75-fold reduction in p-mTOR in WT mice was reduced to 0.5-fold (Figures 3A–C). The levels of BUN and creatinine increased to a greater extent in SIRT3^−/−^ than in WT mice treated with the AMPK inhibitor, ComC, and exposed to CLP, confirming the involvement of AMPK, in the protective effects of SIRT3 (Figures 3D–G). The AMPK inhibition also resulted in lower levels of LC3II and BECN1 and higher levels of cleaved caspase-3 in SIRT3^−/−^ than in WT mice (Figures 3H–K). Immunohistochemical detection of the macrophage marker F4/80 in kidney tissues and the quantification of the result, showed that inhibition of AMPK significantly increased the number of F4/80-positive cells in SIRT3^−/−^ mice compared with those in WT mice exposed to CLP (Figures 3L,M). Similar results were obtained for the levels of the proinflammatory cytokines, TNF-α and IL-1β (Figures 3N,O), indicating that SIRT3 knockout increased inflammatory responses associated with sepsis-induced AKI through the AMPK/mTOR/autophagy pathway.

### SIRT3 Protects Against Kidney Injury by Inducing Autophagy via the AMPK/mTOR Pathway

**FIGURE 4:**
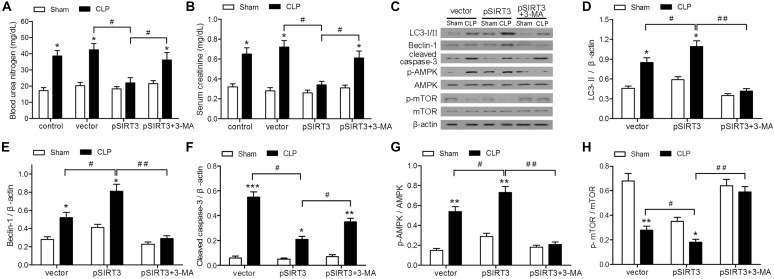
Effect of autophagy inhibition on the protective effect of sirtuin (SIRT)3 against renal function and apoptosis mediated by the AMP-activated protein kinase/mammalian target of rapamycin (AMPK/mTOR) pathway. The C57BL/6 mice were injected intravenously (i.v.) via the tail vein with 100 μl pGC-FU vector (vector) or vector expressing SIRT3 (pSIRT3) lentiviral plasmids at 2 weeks and 24 h before cecal ligation and puncture (CLP) or sham surgery. The pSIRT3 injected mice were treated i.p. with 3-methyladenine (3-MA; 30 mg/kg, pSIRT3+3-MA) or C in equivalent volumes at 12.00 and 1 h before CLP. Kidney tissues and blood samples were collected at 24 h after CLP. **(A)** Blood urea nitrogen (BUN) and **(B)** serum creatinine (SCr) were analyzed using commercial kits. **(C)** Western blotting of LC3-I/II, Beclin-1 (BECN1), cleaved caspase-3, phospho (p)-AMPK, and p-mTOR in the whole kidney. **(D–H)** Relative protein levels were determined after normalization to β-actin, AMPK, or mTOR. All data were expressed as the mean ± SEM (*n* = 6 mice/group). ^∗^*P* < 0.05, ^∗∗^*P* < 0.01, ^∗∗∗^*P* < 0.001 versus Sham in each group. ^#^*P* < 0.05, ^##^*P* < 0.01.

The role of SIRT3 in sepsis-induced AKI was investigated further by overexpressing SIRT3 in mice subjected to CLP or sham surgery. The CLP-induced increase in the levels of BUN and creatinine was abolished in SIRT3-overexpressing mice, whereas treatment with the autophagy inhibitor 3-MA, restored the elevation of BUN and creatinine (Figures 4A,B). Western blotting of autophagy markers and activation of AMPK and mTOR showed that SIRT3 overexpression increased the CLP-induced autophagy, as indicated by elevated levels of LC3II and BECN1; this effect was abolished by 3-MA (Figures 4C–E). Expression of cleaved caspase-3 was significantly lower in SIRT3-overexpressing mice, whereas autophagy inhibition partially restored the levels of cleaved caspase-3 (Figures 4C,F). The CLP-induced increase in the relative levels of p-AMPK and decrease in those of p-mTOR were significantly enhanced by SIRT3 overexpression and suppressed by autophagy inhibition (Figures 4C,G,H). These results suggested that SIRT3 inhibited kidney injury in CLP mice by apoptosis and inducing autophagy in an AMPK/mTOR-dependent manner.

### SIRT3 Overexpression Attenuates Sepsis-Induced Kidney Injury, Apoptosis, and Inflammation by Modulating Autophagy

**FIGURE 5:**
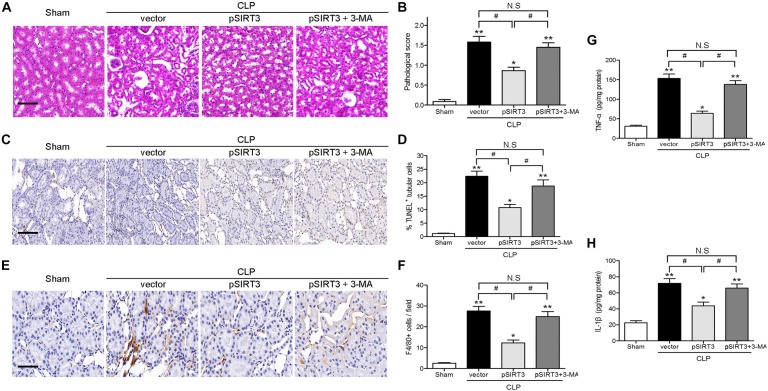
Effect of autophagy inhibition on the protective effect of sirtuin (SIRT)3 against kidney damage, tubular cell apoptosis, and inflammation. The C57BL/6 mice were injected intravenously (i.v.) via the tail vein with 100 μl pGC-FU vector (vector) or the vector expressing SIRT3 (pSIRT3) lentiviral plasmids at 2 weeks and 24 h before cecal ligation and puncture (CLP). The pSIRT3 injected mice were treated i.p., with either 3-methyladenine (3-MA; 30 mg/kg, pSIRT3+3-MA) or C in equivalent volumes at 12.00 and 1 h before CLP. The kidney tissues were collected at 24 h after CLP. **(A)** The collected kidneys of mice were stained with hematoxylin and eosin. Scale bars: 50 μm. **(B)** Quantitative evaluation of morphological tubular damage. **(C,D)** Apoptosis of renal tubular cells of mice was measured by the transferase terminal UTP nick end labeling (TUNEL) assay and quantified. Scale bars: 50 μm. **(E)** F4/80 expression was detected using immunohistochemical staining. Scale bars: 25 μm. **(F)** Number of F4/80^+^ cells per field. Levels of tumor necrosis factor TNF-α **(G)** and interleukin (IL)-1β **(H)** were measured by ELISA in the kidney homogenate. All data were expressed as the mean ± SEM (*n* = 6 mice/group). ^∗^*P* < 0.05, ^∗∗^*P* < 0.01 versus Sham group. ^#^*P* < 0.05.

Apoptosis and inflammation and kidney tissues were stained with H&E, to examine the effect of SIRT3 on tissue injury, and the pathological score was determined. The SIRT3 overexpression decreased the pathological score, whereas this effect was restored by autophagy inhibition (Figures 5A,B). The TUNEL assay and immunohistochemical staining for F4/80 showed that SIRT3 overexpression decreased CLP-induced tubular cell apoptosis and inflammatory responses, whereas inhibition of autophagy with 3-MA restored these effects (Figures 5C–F). Similar results were obtained by ELISA of proinflammatory cytokines, which showed a decrease in TNF-α and IL-1β levels in SIRT3-overexpressing mice exposed to CLP and a reversal to vector control levels, in response to 3-MA treatment (Figures 5G,H). These results indicated that the effects of SIRT3 on attenuating sepsis-induced AKI, tubular cell apoptosis, and inflammatory responses were mediated by induction of autophagy.

## Discussion

In our previous study, we showed that SIRT3 plays a protective role against mitochondrial damage in the kidney by attenuating ROS production, inhibiting the attenuating oxidative stress and downregulating IL-1β and IL-18. Here, we further examined the mechanisms underlying the effect of SIRT3 on sepsis-induced AKI, by focusing on the potential involvement and modulation of autophagy by AMPK and mTOR. AKI was induced by CLP in SIRT3 KO mice; examination of serum and kidney tissues after treatment with selective inhibitors indicated the involvement of autophagy, modulated by activation of AMPK and mTOR, in the protective effects of SIRT3, on kidney function and structure. The SIRT3 overexpression in our mouse model confirmed the involvement of autophagy and its regulating kinases, the AMPK and the mTOR. This study however, had some limitations. Firstly, entire kidney lysates were analyzed; therefore, the specific cell type in which the SIRT3 pathway is most important in this model could not be determined. Secondly, we tested only a limited number of models.

The SIRTs are involved in many cellular processes by targeting a wide array of proteins, and the mitochondrial SIRT3 plays numerous roles in energy production and the maintenance of homeostasis (Rodriguez et al., 2013; Lombard and Zwaans, 2014). Autophagy is an essential pathway for the maintenance of cellular homeostasis and the response to stress; dysregulation of autophagy is involved in the pathogenesis of many diseases (Shibata et al., 2006; Jia et al., 2007; Singh et al., 2009; Lipinski et al., 2010; Rubinsztein et al., 2011). The dysregulation of autophagy pathways has been implicated in renal diseases including AKI, polycystic kidney disease, and diabetic nephropathy (Huber et al., 2012; Kume et al., 2012a,b). The SIRT1 induces autophagy directly by deacetylation of transcription factors that activate autophagy genes (Lenoir et al., 2016). We showed that SIRT3 deletion inhibited autophagy in CLP mice in parallel with increased levels of BUN and creatinine, which indicated renal dysfunction. These effects were accompanied by downregulation of p-AMPK and upregulation of p-mTOR. All the above results emphasize the importance of our findings elucidating a potential mechanism underlying the protective effect of SIRT3 against AKI, which is mediated by induction of autophagy.

Autophagy is regulated by two main nutrient-sensing pathways, mTOR and AMPK (Lenoir et al., 2016). Once activated, AMPK regulates many mitochondrial functions through phosphorylation of downstream effectors, such as peroxisome proliferator-activated receptor γ coactivator (PGC)-1α, SIRT1, and unc-like kinase 1 (ULK1) (Jeon, 2016). The regulatory mechanism of autophagy is complicated and has not been fully elucidated. Type III phosphatidylinositol 3-kinase (PI3K) is required for autophagosome formation (Yao et al., 2015; Du et al., 2016) and its downstream signal AKT, activates BECN1, to increase autophagy levels (Wang et al., 2012). The BECN1 interacts with the antiapoptotic protein Bcl-2 to regulate autophagy and apoptosis (Pattingre et al., 2005; Yang and Yao, 2015). Activation of these signaling pathways induces partial phosphorylation of the autophagy-related gene 13 (Atg13) to bind the autophagy-related gene 1 (Atg1), resulting in the activation of the Atg1, Atg5, and LC3-II that are involved in the formation of autophagosomes and lysosomes (Samara et al., 2008; Won et al., 2015). We found that the formation of autophagosomes was promoted by high expression of BECN1 and LC3-II after H/R treatment. When cells were treated with 3-MA, expression of BECN1 and LC3-II was downregulated, leading to inhibition of autophagy. In addition, expression of p-AMPK was downregulated by the classical class III PI3K inhibitor and 3-MA (Dai et al., 2017). Based on the above results, we concluded that autophagy is associated with AMPK/mTOR, in the CLP mouse model. We showed that SIRT3 knockdown inhibited autophagy in a CLP mouse model in parallel with downregulation of p-AMPK and upregulation of p-mTOR. The overexpression of SIRT3 promoted autophagy concomitant with p-AMPK, upregulation of p-mTOR downregulation and confirmed the involvement of AMPK/mTOR-related autophagy, in the protective effect of SIRT3 against sepsis-induced AKI. It has been reported that SIRT3 knockdown can reduce AMPK phosphorylation, and the ability of SIRT3 to activate AMPK depends on its deacetylase activity (Shi et al., 2010); knockdown of SIRT3 significantly inhibits activation of AMPK kinase. The above results are consistent with the results of this study (Fu et al., 2012). A previous study showed that activation of AMPK attenuates renal ischemia/reperfusion (I/R) injury, which is associated with the development of AKI (Lempiäinen et al., 2012). Treatment of a rat model of kidney I/R injury with 5-aminoimidazole-4-carboxamide nucleotide (AICAR), an activator of AMPK, decreased tubular necrosis, increased p-AMPK/AMPK ratio, and prevented I/R-injury-associated upregulation of SIRT1. In the present study, SIRT3 was used, and similar results were obtained. Targeting AMPK improved several kidney parameters in the study, examining the role of mitochondrial dysfunction in the pathogenesis of sepsis-induced AKI in the model of CLP (Li et al., 2016). In that study, AMPK-treated mice showed an improved glomerular filtration rate and increased expression of autophagy-related proteins, as well as decreased expression of apoptosis-related proteins. The ComC is commonly used as an AMPK antagonist in the AMPK/mTOR pathway (Zhou et al., 2001; Mccullough et al., 2005; Viollet et al., 2010), but there are also reports of inhibition of other kinase activities (Bain et al., 2007; Vogt et al., 2011). In our study, ComC-treated mice showed reduced renal function, confirming our view, that AMPK inhibition can cause kidney damage. Moreover, the autophagy-related protein expression was also consistent with our previous studies. Another recent study examined the role of AMPK-induced autophagy in renal proximal tubular cell death in an *in vitro* I/R injury model and showed that silencing of AMPK promoted mTOR phosphorylation and suppressed autophagy, whereas the mTOR inhibitor RAD001 promoted autophagy and attenuated cell apoptosis during I/R (Wang et al., 2013). These studies support the protective role of autophagy against renal tubular cell injury via the AMPK-regulated mTOR pathway. Similar results regarding the role of SIRT3 were reported in an oxygen and glucose deprivation (OGD) model of neuronal ischemia (Dai et al., 2017), This study suggested that SIRT3 protects against OGD-induced injury by promoting autophagy via the AMPK/mTOR pathway, which supports present results.

At present, autophagy and mitochondrial dysfunction are internationally recognized mechanisms of sepsis-induced AKI. In our previous studies, SIRT3 protected the kidney mitochondria, and SIRT3 has been confirmed in this study. The AMPK/mTOR can induce autophagy, which may be the first stage in the mechanism of sepsis-induced AKI.

## Conclusion

In conclusion, this study uncovered a potential mechanism underlying the protective role of SIRT3 against sepsis-induced AKI, that involved induction of autophagy through the AMPK/mTOR pathway. Further investigation is needed to elucidate the role and identify targets of deacetylation in the effect of SIRT3, and to further examine inflammatory factors in the induction of autophagy. The present findings are important in supporting the AMPK/mTOR pathway and SIRT3 as therapeutic targets for AKI.

## Author Contributions

WZ, LZh, and LZe designed the research, analyzed the data, and drafted the manuscript. WZ, RC, and HL performed the experiments. LZh, MS, and YZ helped with data acquisition and discussion. LZh and YZ analyzed the data and prepared the figures. LZe supervised the whole project. All authors contributed to manuscript writing and editing.

## Conflict of Interest Statement

The authors declare that the research was conducted in the absence of any commercial or financial relationships that could be construed as a potential conflict of interest.
